# Posterior Cloacal Variant with Clitorolabial Transposition and a Rectoperineal Fistula

**DOI:** 10.1055/s-0041-1728724

**Published:** 2021-05-18

**Authors:** Niveshni Maistry, Giulia Brisighelli, Chris Westgarth-Taylor

**Affiliations:** 1 Department of Paediatric Surgery, Chris Hani Baragwanath Hospital, Diepkloof, Soweto, Gauteng, South Africa

**Keywords:** posterior cloacal variant, anorectal malformation, cloaca

## Abstract

We present a case and discuss the management of a posterior cloacal variant not as yet described in the literature. A 5-week-old infant presented to our institution with a posterior cloacal variant and transposition of the clitoris and labia. After initial radiological investigations, staged operative intervention was performed over a 1-year period. This included an initial laparotomy (with drainage of hydrocolpos and formation of a colostomy), a left ureteric reimplantation and a posterior sagittal anorectoplasty due to a rectoperineal fistula. The child is under continued long-term follow-up by our specialist pediatric surgical team.

## Introduction

The first case of a posterior cloaca was reported in 1991. 1 Since then, only 39 additional cases have been reported in the literature, making it an incredibly rare congenital malformation. 2 3 4 5 We present a case of a posterior cloacal variant associated with clitorolabial transposition that underwent complex multidisciplinary management at our institution.

## Case Report

A 5-week-old infant was referred to our tertiary institution from a rural setting with an initial suspicion of a disorder of sexual differentiation due to abnormal external genitalia.

**Fig. 1:**
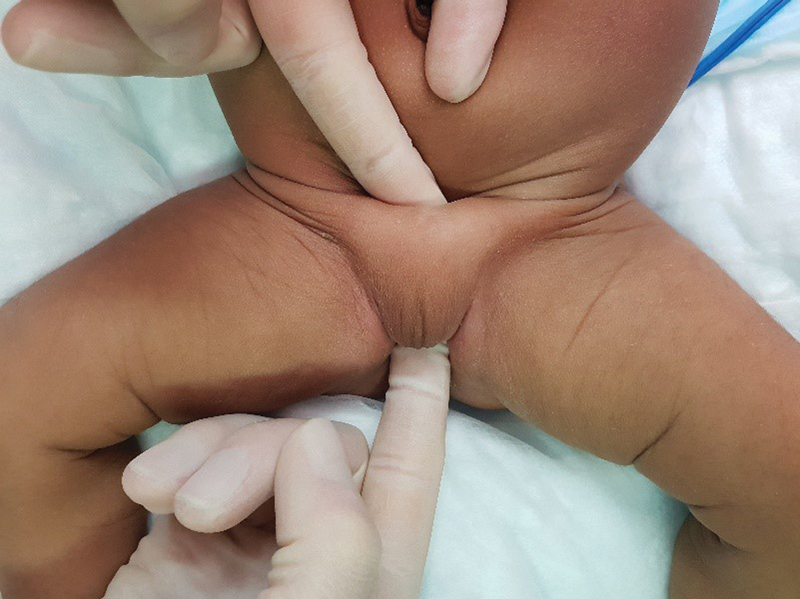
Demonstration of thickened pubic symphysis.

**Fig. 2:**
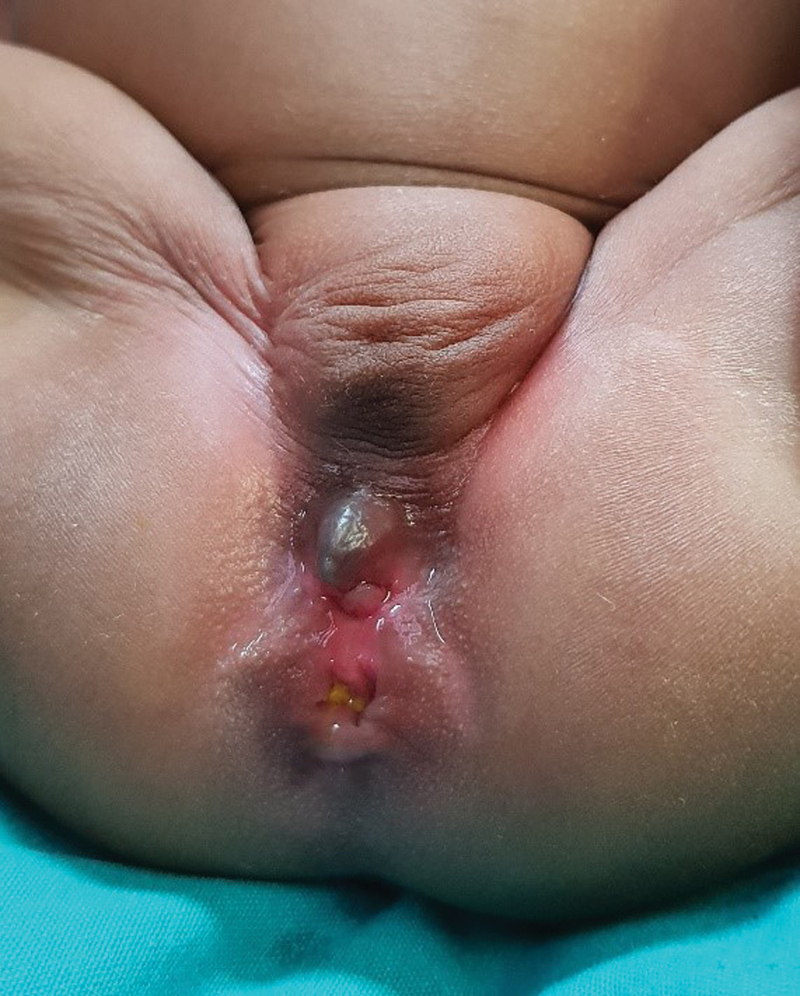
External appearance of perineum and genitalia.

Initial examination revealed a clinically well infant with fused superior labia and an inferiorly displaced clitoris. The child was noted to have a thickened pubic symphysis measuring ∼3 cm ( Fig. 1 ). Examination of the perineum demonstrated two openings: a rectoperineal fistula and a common channel opening directly anterior to it in the midline ( Fig. 2 ). The patient was passing stools without straining and the rectoperineal fistula admitted a size 12 Hegar dilator with ease. After stretching the labia to better visualize the perineum, vaginal and urethral orifices were not separately discernible within the common channel. Apart from mild frontal bossing, no other facial or skeletal dysmorphic features were noted.

Laboratory investigations confirmed a 46XX karyotype with a normal endocrine work-up. Renal dysfunction was noted with an elevated urea and creatinine but with normal electrolytes. An abdominal ultrasound revealed a small echogenic right kidney, a bulky echogenic left kidney with gross left hydroureteronephrosis, and a complex pelvic fluid collection suspicious for hydrocolpos. X-rays demonstrated a normal sacrum.

A urinary catheter was placed into the urogenital sinus and clear urine was drained. The renal dysfunction improved with the administration of intravenous fluids.

Ultrasound was not able to confirm whether the balloon of the catheter was indeed within the bladder or within the vagina. As a result, the decision was made to take the child for an examination under anesthesia and perineal stimulation. The anal opening was confirmed to be significantly anterior to the sphincter complex. A laparotomy was therefore performed to fashion an end colostomy and drain the hydrocolpos. At laparotomy, a normal uterus and fallopian tubes were identified and the vagina appeared thickened and hypertrophic without significant fluid content. A formal vaginostomy was therefore not a feasible option as the vagina was not dilated enough to reach the abdominal wall. Hence, a vaginotomy was done and a catheter was inserted via the common channel into the vagina under direct vision.

Unfortunately, the child developed wound sepsis and sheath dehiscence and required a relook laparotomy and sheath closure 4 days after the initial surgery. The child recovered well and a repeat abdominal ultrasound 10 days postoperatively revealed resolved hydrocolpos but residual left-sided hydronephrosis.

A cystovaginoscopy was performed at 3 months of age. The common channel measured 1.5 cm. The urethra measured 3.5 cm with a normal bladder neck. An adequate vagina with a normal cervix was also demonstrated. On entering the bladder, only the right ureteric orifice was visualized.

A mercaptoacetyltriglycine renogram was completed which revealed a nonfunctional right kidney, with slow drainage from a hydronephrotic left kidney. A voiding cystourethrogram demonstrated Grade 5 vesicoureteric reflux on the left.

Repeat cystoscopy with a STING (subureteral teflon injection) procedure was therefore attempted—this was however unsuccessful as the left ureteric orifice was unable to be visualized. Throughout this time, the child was regularly seen by the pediatric renal unit who monitored her renal function which continued to remain normal.

At 11 months of age, the patient underwent an uncomplicated left cross trigonal Cohen's ureteral reimplantation and recovered well postoperatively. The plan was to concomitantly perform a posterior sagittal anorectoplasty (PSARP). This was however postponed due to a severe nappy rash.

**Fig. 3:**
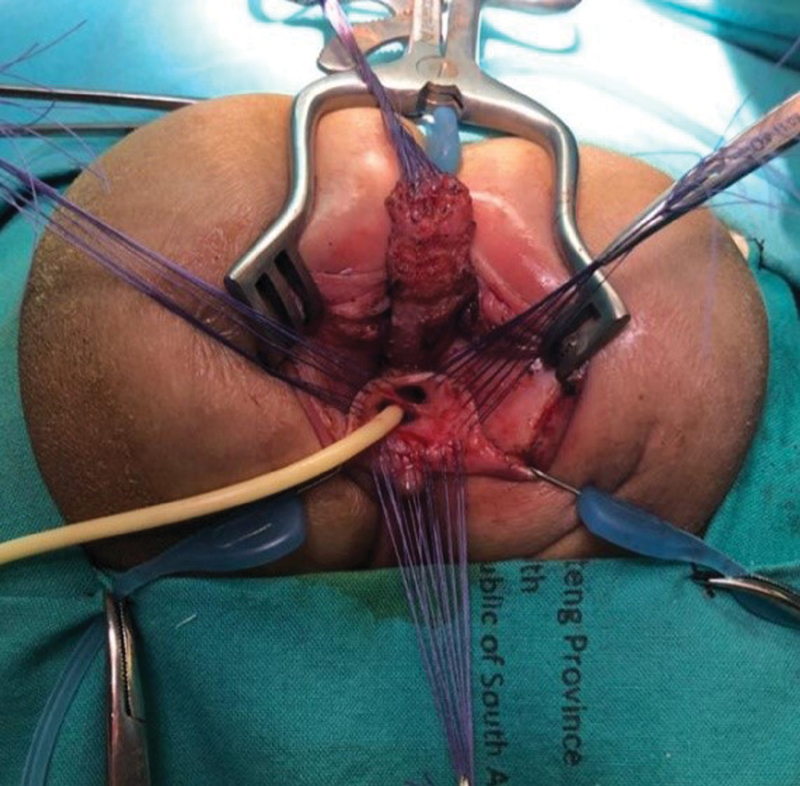
Intraoperative image: posterior sagittal anorectoplasty and total urogenital mobilization.

**Fig. 4:**
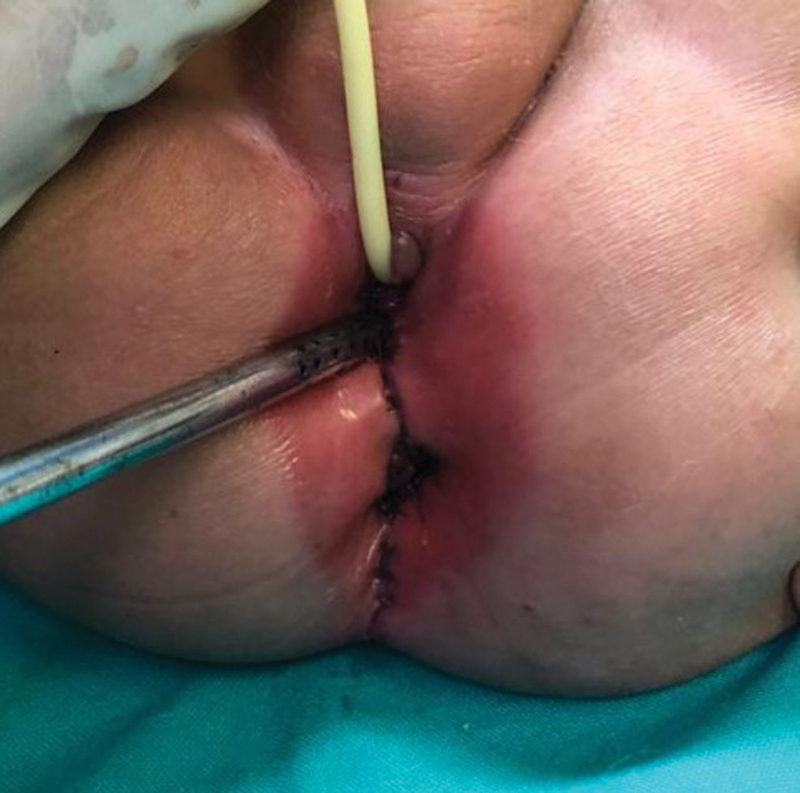
Postoperative image: urinary catheter in urethra, suction catheter in vagina, and neoanus at inferior margin.

At 1 year of age, the patient underwent a PSARP along with a total urogenital mobilization ( Figs. 3 and 4 ).

The patient recovered well postoperatively. She was discharged with anal dilations being performed by her caregiver. She is followed up regularly. Her colostomy closure was successfully performed at the age of 20 months (delayed due to the coronavirus pandemic). She is under continued renal follow-up and plans for a genitoplasty to correct the transposition of the clitoris and labia will be made going forward.

## Discussion

Posterior cloacas and posterior cloacal variants are incredibly rare congenital malformations. They were initially referred to as type A and type B malformations, 2 but the terminology was refined to posterior cloacas and posterior cloacal variants after recognizing that the anomaly exists within a spectrum of presentations. 3 Approximately 35% of reported cases are variants. Posterior cloacas may be mistaken for a urogenital sinus with a normal anus. This can be differentiated by the placement of the urogenital sinus, which is more posterior in patients with a posterior cloaca, and distant from the location of the normal urethral opening. Patients with posterior cloacas have a normally placed anus, which has good implications for bowel control as the patient ages. Patients with posterior cloacal variants may have abnormally sited anuses, which may lead to poorer bowel control outcomes. 3

Our patient displayed many similarities to previously published cases. Urological anomalies have been shown to be present in 88% in reported cases. Of these, 34% have had unilateral reflux and 31% have had a single/nonfunctional kidney. 2 3 4 Hydrocolpos was found to be present in 65% of posterior cloacal cases—significantly more than in other cloacal forms. This is believed to be as a result of the unique anatomic configuration which limits fluid drainage from the vaginal opening. 3 It is therefore imperative to always suspect, diagnose, and treat hydrocolpos in these patients. This can be done with intermittent catheterizations or with a formal vaginostomy. In our setting, because of poor socioeconomic circumstances, we believe the second option to be safer.

Pubic symphysis hypertrophy is almost universally present. This frequently necessitates carving of the pubic bone and cartilage during reconstructive surgery. 3 4 This was not required in our patient since there was adequate space for operative intervention and sufficient domain for pelvic structures. The exact reason for the occurrence of the hypertrophy is unknown, but it speculated to be because of the abnormally posterior development of the urogenital sinus. It could however also be the inverse, in that the urogenital sinus may be forced to develop in a more posterior position because of an abnormally large pubic symphysis. No conclusive evidence exists.

Although there are documented cases of posterior cloacal variants associated with abnormal external genitalia, 4 5 to our knowledge, there are no reported presentations with clitorolabial transposition. The primary reason for referral to our unit was the abnormal genitalia which lead to the detection of the posterior cloacal variant. As a result of the normally sited anus in posterior cloacas (or in the case of the variants, a rectoperineal fistula that is usually well sized), these patients can often have very delayed presentations.

The surgical recommendation based on the literature is to, wherever possible, avoid mobilization of the anus (if the anus is correctly placed within the sphincter). This is to avoid damaging the muscles and the nerve supply which would in turn give the child the best outcome in terms of continence. Transperineal or transanorectal total urogenital mobilization is therefore the preferred approach. 2 3 The decision to perform a PSARP in our patient was based on the fact that the anus was located anterior to the anal musculature demonstrated with stimulation.

As yet, there is no definitive evidence/guidelines that exist for the management of posterior cloacal variants.

## Conclusion

The surgical management of posterior cloacal variants and their associated anomalies is complex and is best handled by a specialist pediatric colorectal surgical team. The presence of hydrocolpos always needs to be suspected, investigated, and treated. A PSARP should only be performed in the case the anus is outside of the sphincter complex. Clinical and surgical management should be tailored to individual patients according to the anatomy of the perineum and presence of associated anomalies.

## Notes

New Insights and the Importance for the Pediatric Surgeon The surgical management of rare complex anorectal malformations is incredibly challenging. The description of our utilized management strategies may offer new insights/perspectives to pediatric surgeons across the globe.
